# A Mysterious Cause of Coagulopathy With Bleeding Diathesis

**DOI:** 10.7759/cureus.76129

**Published:** 2024-12-21

**Authors:** Mónica Baptista Lopes, Carolina Saca, Ricardo Paquete Oliveira, Ana Bastos Furtado, José Delgado Alves

**Affiliations:** 1 Department of Internal Medicine IV, Hospital Professor Doutor Fernando Fonseca, Amadora, PRT

**Keywords:** blood coagulation disorders, drug poisoning, spontaneous hemorrhage, superwarfarins, vitamin k antagonists (vkas)

## Abstract

Vitamin K is essential to produce functional vitamin K-dependent coagulation factors (prothrombin, factors VII, IX, and X). Vitamin K antagonists inhibit the normal activation of these factors, leading to bleeding manifestations of variable severity. Long-acting vitamin K antagonists or superwarfarins were developed as rodenticides and have a significantly longer half-life and greater potency when compared to warfarin. Intoxication with superwarfarins presents a typical clinical picture and evolution, but its diagnosis can be challenging without a clear ingestion history. We describe a case of a healthy 67-year-old man who presented with bleeding diathesis and severe anemia due to isolated coagulopathy. Laboratory evaluation showed prolonged coagulation times and very low levels of vitamin K-dependent coagulation factors. All other potential causes were ruled out, and superwarfarin intoxication was assumed, although we were unable to confirm unequivocal exposure. He was treated with high-dose vitamin K supplementation for two months until coagulation times normalized. He remained asymptomatic with no laboratory changes four months after the suspension of vitamin K.The management of a patient presenting with bleeding diathesis and coagulopathy can be challenging. A stepwise approach is helpful to reach the correct diagnosis and intoxication with long-acting vitamin K antagonists should be considered, even without an ingestion history. Therapy with vitamin K in high doses must be started as soon as possible and can be lifesaving.

## Introduction

Vitamin K is essential for the post-translational carboxylation of selected glutamic acid residues in the precursor forms of vitamin K-dependent proteins, such as prothrombin, factor VII, factor IX, and factor X. This modification enables these factors to function normally. This carboxylation is catalyzed by the enzyme gamma-glutamyl carboxylase (GGCX) and results in the conversion of the vitamin K hydroquinone to vitamin K epoxide. The latter must then be recycled back to its reduced form, completing the so-called vitamin K cycle, in a reaction catalyzed by the enzyme vitamin K epoxide reductase (VKOR) [1].

Vitamin K antagonists, such as warfarin, are VKOR inhibitors that block the reconversion of vitamin K to its reduced form, disrupting the cycle and preventing the normal activation of vitamin K-dependent coagulation factors [2,3]. Long-acting vitamin K antagonists, also known as superwarfarins, such as brodifacoum, difenacoum, and bromadiolone, are anticoagulant rodenticides derived from warfarin. Their modified chemical structure is responsible for their longer half-life and greater potency compared to warfarin [2,3]. Intoxication with vitamin K antagonists can lead to severe coagulopathy and associated life-threatening bleeding manifestations. Differential diagnosis in patients presenting with these changes includes vitamin K malabsorption and surreptitious intake of other vitamin K inhibitors [2]. Patients intoxicated with superwarfarins have a characteristic clinical and laboratory evolution, however, diagnosis is challenging in the absence of a clear ingestion history and its delay can increase the related morbidity and mortality [3].

## Case presentation

A 67-year-old man presented to our hospital with a sudden onset of macroscopic hematuria, with no other associated symptoms. He had a known history of arterial hypertension and psoriasis, but he was not taking any regular medication for them. He also had a history of long-term alcohol consumption and a current consumption of approximately 120 g of alcohol per day. He had no relevant family history.

Clinical examination was remarkable only for hematuria with clots and bleeding gums. There were no other signs of bleeding diathesis. Laboratory results showed an activated partial thromboplastin time (aPTT) of 141 seconds, and a prothrombin time (PT) and international normalized ratio (INR) prolonged beyond the measured limits of 100 seconds and 10, respectively. There were no other significant findings, namely anemia, thrombocytopenia, fibrinogen consumption, elevated D-dimers, or signs of renal or hepatic dysfunction (Table 1). Mixing tests led to the correction of the clotting times. The patient was administered 10 mg of intravenous vitamin K with no correction of the clotting times. He was admitted for further workup and clinical surveillance. 

**Table 1:** Patient's laboratory results at admission MCV, mean corpuscular volume; PT, prothrombin time; INR, international normalized ratio; aPTT, activated partial thromboplastin time.

Parameter	Patient values	Reference range
Hemoglobin (g/L)	12.7	13.0-17.0
MCV (fL)	92.3	78.0-96.0
Leucocytes (x10^9^/L)	9.0	4.0-10.0
Platelets (x10^9^/L)	205	150-410
PT (seconds)	>100	9.7-11.8
INR	>10	<1.2
aPTT (seconds)	141.0	20.6-29.5
Fibrinogen (g/L)	6.7	1.8-3.5
D-dimers (mcg/L)	437	<500
Sodium (mmol/L)	136	136-145
Potassium (mmol/L)	4.85	3.5-5.1
Chloride (mmol/L)	98.6	98-107
Calcium (mg/dL)	8.8	8.8-10.2
Phosphate (mg/dL)	2.6	2.5-4.5
Magnesium (mg/dL)	2.2	1.6-2.6
Aspartate aminotransferase (U/L)	24	<40
Alanine aminotransferase (U/L)	14	<41
Alkaline phosphatase (U/L)	59	40-130
Gamma-glutamyl transpeptidase (U/L)	29	<60
Total bilirubin (mg/dL)	0.3	<1.2
Lactate dehydrogenase (U/L)	276	135-225
Creatinine (mg/dL)	1.06	0.7-1.2
Urea (mg/dL)	36	<50
Total serum protein (g/dL)	6.75	6.4-8.3
Albumin (g/dL)	4.2	3.97-4.94
Creatine kinase (U/L)	112	39-308
C-Reactive protein (mg/dL)	0.84	<0.5

Within the first 24 hours after admission, the patient presented an episode of hematemesis and his hemoglobin dropped to a minimum of 6 g/dL. Consequently, he was transfused with two units of packed red blood cells and four units of fresh frozen plasma (FFP), which corrected the coagulopathy. Following this, the hematuria and bleeding of the gums resolved, and there were no further episodes of gastrointestinal bleeding. An endoscopy performed at this time showed only an erosive duodenopathy and a small duodenal ulcer with no active bleeding. The hemoglobin level remained stable around 9 g/dL thereafter. 

Approximately 24 hours after the infusion of FFP, there was a recrudescence of the coagulopathy (PT 87.5 seconds, INR 9 and aPTT 37.6 seconds). Given the more pronounced PT prolongation compared to the aPTT, vitamin K supplementation with phytomenadione was tried once more, this time with higher doses (intravenous 20mg/day). This led to a gradual correction of the clotting times, reaching a minimum INR of 1.2. However, every time the vitamin K was stopped, the coagulopathy resumed within 24 hours. Dosing results of coagulation factors evaluated at this point showed very low levels of prothrombin (29%), factor VII (12%), factor IX (22%), and factor X (18%), while factor V (209%), factor VIII (>480%) and factor XI (107%) were elevated (Table 2).

**Table 2:** Results of the dosing of coagulation factors

Coagulation factor	Patient values (%)	Reference range (%)
Prothrombin	29	70-120
Factor V	209.8	70-120
Factor VII	>130	50-130
Factor VIII	>480	70-150
Factor IX	3	70-120
Factor X	17	70-120
Factor XI	107	70-120

The observed deficiency of vitamin K-dependent coagulation factors and the correction of the altered clotting times with high doses of vitamin K suggested a vitamin K deficiency, possibly due to reduced ingestion or impaired absorption. The patient exhibited no signs of malnutrition, had normal serum albumin and total protein levels, and lacked other frequent nutritional deficiencies such as iron, cyanocobalamin, or folic acid. Dosing of liposoluble vitamins showed normal levels with vitamin E at 979 mcg/dL (normal range 500-2,000 mcg/dL) and vitamin K at 2.8 ng/mL (normal range 0.1-3 ng/mL), but reduced levels of vitamin A at 20 mcg/dL (normal range 30-70 mcg/dL) and vitamin D at 9 ng/mL (normal range 30-100 ng/mL). Furthermore, the patient did not have any gastrointestinal symptoms suggestive of malabsorption (such as diarrhea or steatorrhea). Celiac disease serologies were also negative and parasitological examination of the stool showed no eggs, cysts or parasites. Lastly, the abdominal ultrasound and abdominal and pelvic computed tomography (CT) scan were normal.

Lastly, we considered the possibility of the ingestion of a long-acting vitamin K antagonist. Reviewing the patient’s social background, we discovered that he was unemployed and was being evicted from his apartment. Most of his meals were prepared by him, but occasionally he consumed food offered by friends, that was usually left in the street outside his apartment. He denied any voluntary consumption of drugs, medications or chemical products and also denied having any poisons stored in his apartment. Unfortunately, toxicologic studies were not feasible at this time, as it was more than two weeks after his admission. Nevertheless, we decided to assume this diagnostic hypothesis, as it was the only one that explained the patient’s clinical presentation and response to vitamin K supplementation. 

He was maintained on high doses of vitamin K, initially administered intravenously (20 mg/day) and, after achieving only slightly elevated INR levels (1.5-2), orally (40 mg/day). He was discharged 25 days after admission, with programmed weekly assessments at our ambulatory clinic. After discharge, he maintained near normal INR levels (1-1.5), which allowed a reduction in oral vitamin K dose to 20 mg/day. The supplementation was stopped five weeks after discharge (approximately nine weeks after initial admission). Clinical and laboratory evaluations conducted two weeks and four months after stopping vitamin K supplementation showed no bleeding symptoms and normal clotting times (PT 11.6 seconds, INR 0.9 and aPTT 28.6 seconds). 

## Discussion

We present a case of severe isolated coagulopathy with associated bleeding manifestations of an unknown cause, which prompted us to manage the case using a stepwise approach to achieve the correct diagnosis. This patient initially presented with significantly prolonged coagulation times, with a slightly more prolonged TP compared to the aPTT. This discrepancy became more evident when the coagulopathy recurred after the administration of FFP, which was only used as recommended to correct the coagulopathy when the patient evolved with more severe bleeding and acute anemia [2,3]. At this point, mixing tests led to the correction of the coagulation times, which was suggestive of a deficiency of coagulation factors rather than the presence of inhibitors. Additionally, no other clinical or laboratory findings suggested alternative causes of severe coagulopathy, such as disseminated intravascular coagulopathy (DIC). The plasma levels of all the vitamin K-dependent coagulation factors were very low. 

At this stage, differential diagnosis was limited to vitamin K deficiency, the presence of a vitamin K antagonist, or a hereditary deficiency of any of the enzymes involved in the vitamin K cycle (GGCX or VKOR) [2]. The latter was promptly excluded, given that the patient was 67 years old, had been previously healthy, and denied any kind of bleeding symptoms before the present episode. Additionally, his last known laboratory tests were from eight years before this admission and showed no coagulation abnormalities. 

Isolated vitamin K deficiency is rare in the developed world since the daily requirement for this micronutrient is very low (about 100 mg) [2]. Severe malnutrition, however, can lead to vitamin K deficiency, but other signs would typically be present. Despite the patient’s precarious social situation, he was not underfed; his total protein and albumin levels were normal and there were no other common nutritional deficiencies.

Vitamin K is a liposoluble vitamin usually absorbed in the intestines. Gastrointestinal disorders that impair normal nutrient absorption can lead to vitamin K deficiency, even with adequate intake [2]. Conditions such as pancreatic insufficiency, sprue, short gut syndrome, and prolonged use of broad-spectrum antibiotics are some examples of these disorders [2]. Although the patient did not have any gastrointestinal symptoms, we excluded the possibility of celiac sprue or parasitic infections. Eventually, the dosing of all the liposoluble vitamins showed that there was no vitamin K deficit, thereby excluding both the hypothesis of insufficient ingestion and impaired absorption as potential causes. 

Decreased activity of vitamin K-dependent proteins can be observed in liver disease, resulting from the overall decrease in protein synthesis [2]. Despite the patient’s history of alcohol consumption, he did not present any other findings suggestive of liver failure. His albumin and bilirubin levels were normal, all the liver enzymes were within normal ranges, and fibrinogen and its degradation products were also unaltered. The abdominal ultrasound and CT scan revealed a normal liver, and the endoscopy did not show any signs of portal hypertension, which would be expected in the presence of a prolonged liver disease.

At this point, the most plausible hypothesis to explain the case was the presence of a vitamin K antagonist, either a pharmacologic agent or a rodenticide, which is the most common cause of isolated deficiency of vitamin K-dependent coagulation factors [2]. The patient did not have any clinical indication of undergoing anticoagulation and had no active prescriptions nor immediate access to such drugs. He also denied voluntary consumption of any type of anticoagulants in any other context. Furthermore, his clinical evolution, characterized by rapid recrudescence of the coagulopathy 24 hours after FFP administration and every time vitamin K supplementation was stopped, was not consistent with intoxication with pharmacologic forms of vitamin K antagonists [3].

Superwarfarins were developed to overcome increasing resistance to warfarin in rodent populations due to VKOR gene mutations [1,2]. These highly lipophilic drugs concentrate in the liver leading to a high distribution volume, slow elimination rates, and consequently very prolonged anticoagulant effects [4]. This translates into serum half-lives of more than 15 days for all the agents included in this category [1,4,5]. 

Measuring the serum vitamin K and warfarin levels can be useful to establish the diagnosis of superwarfarin intoxication [2]. However, the identification of warfarin does not rule out the concomitant presence of a superwarfarin [6]. Toxicologic studies to identify a superwarfarin in the serum are the test of choice to confirm this diagnosis, but they are often expensive and not always available [2,3,6,7]. Additionally, toxicologic panels may not target the culprit agent since it is rarely possible to elicit that information from the patient [2]. This means that, even when these studies fail to detect one of the more common superwarfarins (such as brodifacoum or bromadiolone), less common similar agents might still be present [2]. Lastly, the disruption of the vitamin K cycle caused by these agents can exist far beyond the detected serum levels [2,4]. Thus, toxicologic testing, while helpful is not strictly required to establish the diagnosis or for subsequent patient management [2]. Considering all this, although we were unable to unequivocally identify the presence of a superwarfarin in this case, we are confident that this remains the only plausible explanation for the clinical and laboratory findings and evolution of this patient.

The source of intoxication is not always evident. Superwarfarin ingestion has been reported in various situations, either accidentally or voluntarily, the latter mainly in suicidal patients [2,3,5,7]. There are also reports of voluntary intake of superwarfarins in combination with drugs of abuse, to prolong their effects (due to saturation and inactivation of liver enzymes common to both agents’ metabolisms), as well as reports of contamination of synthetic recreational drugs with these vitamin K antagonists [3,8]. If the patient’s detailed history does not provide a logical explanation, psychiatric evaluation must be ordered. If self-harm is excluded, collaboration with social services or law enforcement authorities should be considered [2,3].

Although our patient did not undergo a formal psychiatric evaluation, he had no previous history of self-harm, exhibited no abnormal behavior during his prolonged hospital stay, and had no similar episodes during follow-up after discharge. He also denied consumption of any illicit drugs, namely cannabinoids. Considering his previous precarious living situation, we considered the hypothesis of surreptitious contamination of the food he ate that was occasionally left unattended at his doorstep. However, a severe coagulopathy like this would typically require significant or repeated exposure to a superwarfarin [5]. Social services evaluated the case, and after discharge, he was integrated into a community center with supervised meals and medication. Additionally, the ongoing litigious eviction process led us to consider the remote possibility of criminal intent. Given this, the case was reported to the competent authorities, but we are unaware of any subsequent follow-up. 

Treatment must be initiated based on the suspicion of superwarfarin intoxication unless there is a clear history of ingestion [2,3]. High doses of vitamin K supplementation are required because the smaller doses that are traditionally given to manage supratherapeutic warfarin intake are insufficient to overcome the profound suppression of the vitamin K cycle caused by a superwarfarin [3]. This explains why the initial vitamin K infusion was ineffective in our patient. The intravenous route is preferred if bleeding is present, due to its faster effect, but oral formulations are equally effective [2]. 

The optimal dose of vitamin K necessary to rescue the blockade of VKOR is variable and must be determined empirically for each patient via titration using the PT or INR [2,6]. Reported initial doses range from 20 to 400 mg/daily [2]. Once normal or near normal PT values are achieved, vitamin K can be slowly tapered as soon it is no longer needed to maintain normal coagulation times and then discontinued [2]. This can extend over months and sometimes more than a year [2]. Monitoring superwarfarin levels at admission and 24 to 48 hours afterwards may help estimate the duration of treatment, however, as stated earlier, this is often not feasible [3,6]. Our patient needed supplementation for nine weeks following the initial presentation, which is consistent with these timings.

Superwarfarin intoxication is probably more frequent than reported. These are easily obtainable agents and patients often do not disclose their ingestion. Exposure to these toxic agents may result in none or minimal bleeding manifestations, and dosing is frequently not feasible [2,5]. In 2022, in the United States, there were over 3,000 reported cases of intoxication with this type of rodenticides requiring treatment [9]. Similar data is lacking in Portugal, making it difficult to gauge the extent of the problem in our country. As far as we know, to date, only one case of superwarfarin intoxication has been reported, where the patient overtly admitted the intake of bromadiolone at admission, simplifying the diagnostic approach [7]. Our case highlights the importance of raising awareness to this type of intoxication, particularly in the absence of a known ingestion history of toxic agents, as this is often the scenario. Furthermore, treatment is relatively straightforward and should not be delayed as it can be lifesaving in patients presenting with severe bleeding manifestations.

## Conclusions

The diagnostic approach of a patient presenting with isolated coagulopathy can be challenging and intoxication with long-acting vitamin K antagonists should be considered, even in the absence of a clear ingestion history. Initial clinical and laboratory findings alone may be enough to make this diagnosis, given the difficulty in identifying the culprit agent. Treatment with high doses of vitamin K should be initiated as soon as possible in high doses and continued as long as needed to maintain normal or near-normal coagulation times. This simple supplementation is usually readily available and can be lifesaving in patients with severe bleeding symptoms.
